# Supplementary material for: Analysis of key genes in Mycobacterium ulcerans reveals conserved RNA structural motifs and regions with apparent pressure to remain unstructured
Source: Front Trop Dis. Author manuscript; Available in PMC 2023 Mar 30. (PMC10062443; doi:10.3389/fitd.2022.1009362)
Supplement: All Zipped Supplemental Material — SUPPLEMENTARY FIGURE 1 All Mul_RS04200 ScanFold results. Figure showing ScanFold results for Mul_RS04200 including z-score, MFE, ED, base pair diagram, gene cartoon, and 2D model of the structure with a z-score <−2. SUPPLEMENTARY FIGURE 2 All Mul_RS09540 ScanFold results. Figure showing ScanFold results for Mul_RS09540 including z-score, MFE, ED, base pair diagram, gene cartoon, and 2D model of the structure with a z-score <−2. SUPPLEMENTARY FILE S1 M. ulcerans genomic data used in IGV-ScanFold. This file contains the M. ulcerans bacterial genome fasta, virulence plasmid fasta, and their associated gff3 genome annotations. SUPPLEMENTARY FILE S2 All cm-builder covariation data. This file contains all the data required to run cm-builder, all the output files generated by INFERNAL and R-Scape, and results of power analysis. SUPPLEMENTARY FILE S3 OligoWalk and 18-mer ScanFold bar charts. This file contains the OligoWalk and 18-mer partitioned ScanFold data as bar charts overlaid against the gene cartoon for all six genes studied. SUPPLEMENTARY FILE S4 OligoWalk and 18-mer ScanFold raw data. This file contains the raw output data from OligoWalk and in-house script for portioning 18-mer ScanFold data for all six genes of interest. SUPPLEMENTARY FILE S5 All ScanFold-Scan data. This file contains two folders with results from mono- and dinucleotide shuffling for each gene studied. These folders contain the raw ScanFold-Scan output data such as per nucleotide MFE, ED, z-score, input, and output fasta files, and out file. SUPPLEMENTARY FILE S6 All ScanFold-Fold data. This file contains two folders with results from mono- and dinucleotide shuffling for each gene studied. These folders contain the raw ScanFold-Fold output data such as the log file, base pair track, final partners data, all dot bracket files, all CT files, extracted structures gff3 file, and the global VARNA 2D model. [file NIHMS1880345-supplement-All_Zipped_Supplemental_Material.zip › Supplementary Data/Supplementary File S3.pdf]

Mul_RS01365 Anti-sense Oligo Metrics

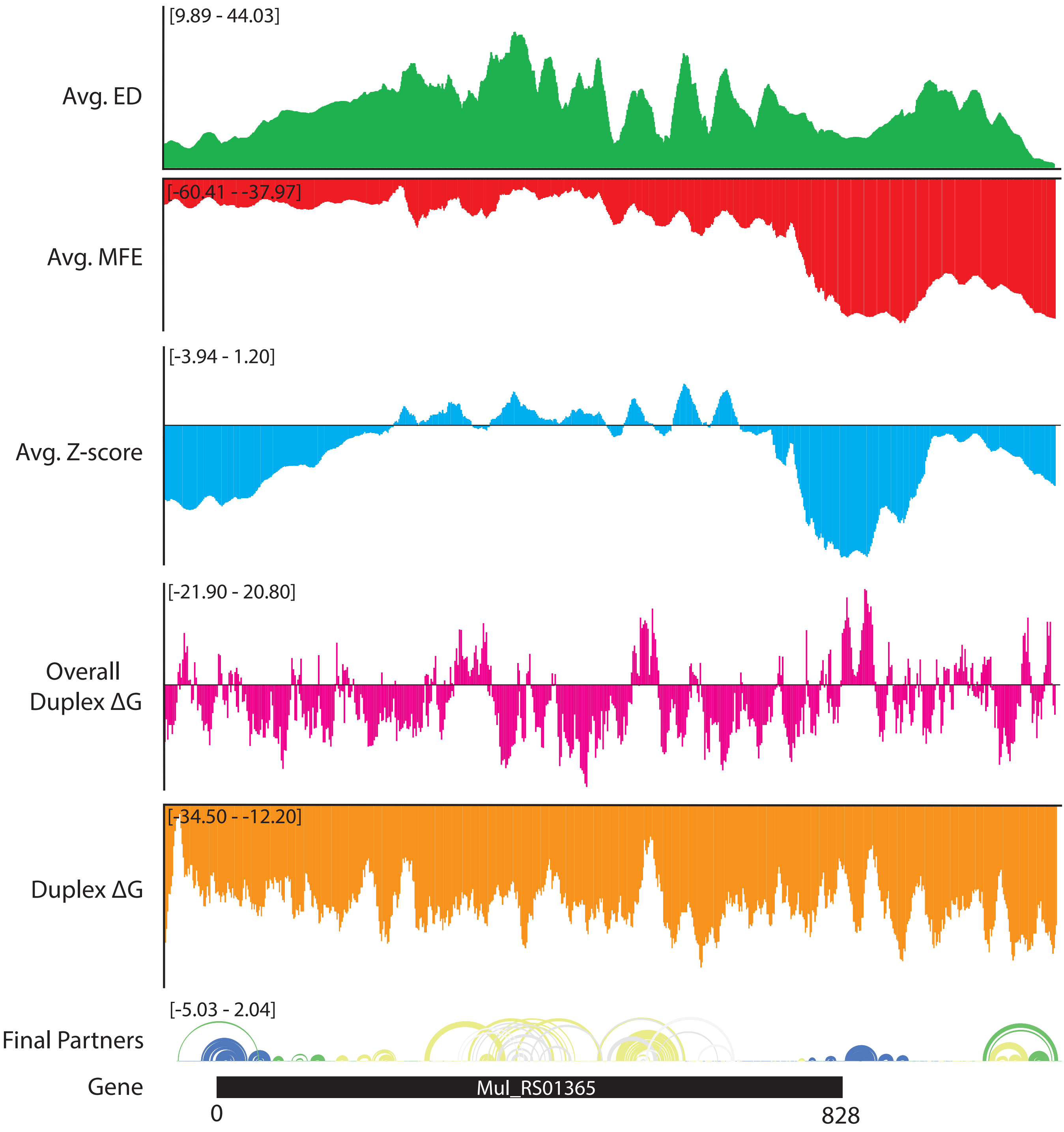

Mul_RS04730 Anti-sense Oligo Metrics

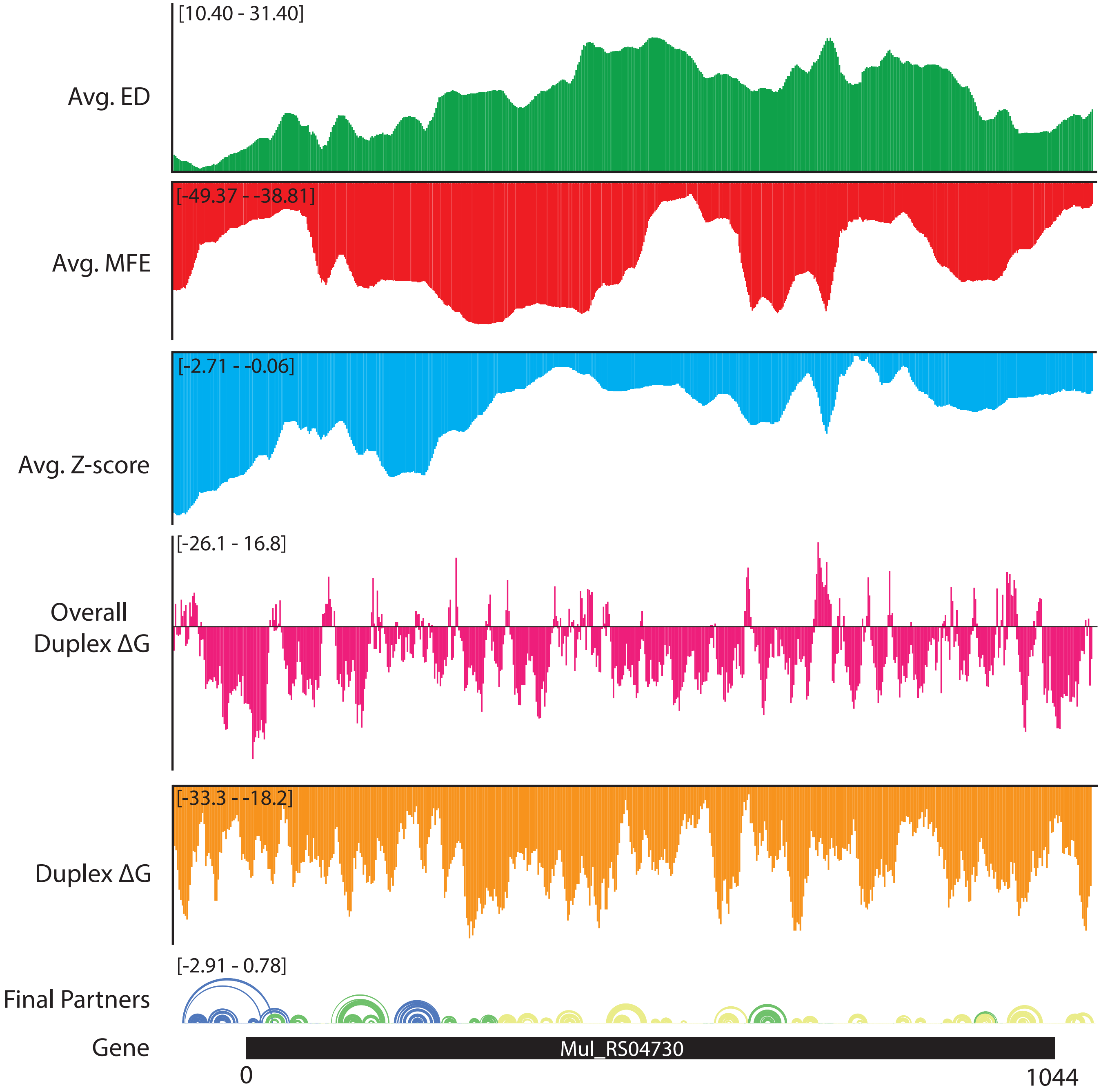

Mul_RS04200 Anti-sense Oligo Metrics

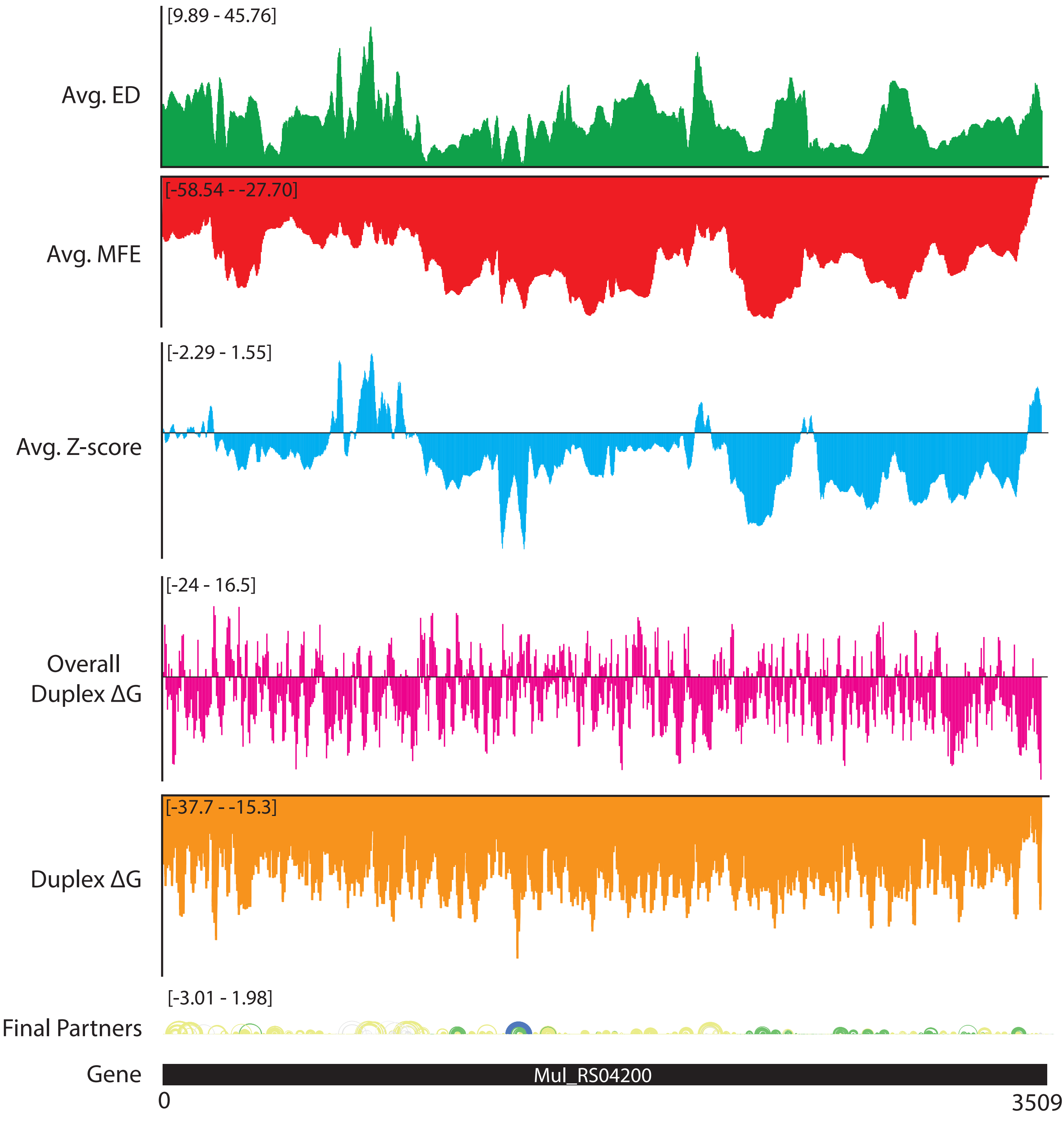

Mul_RS09540 Anti-sense Oligo Metrics

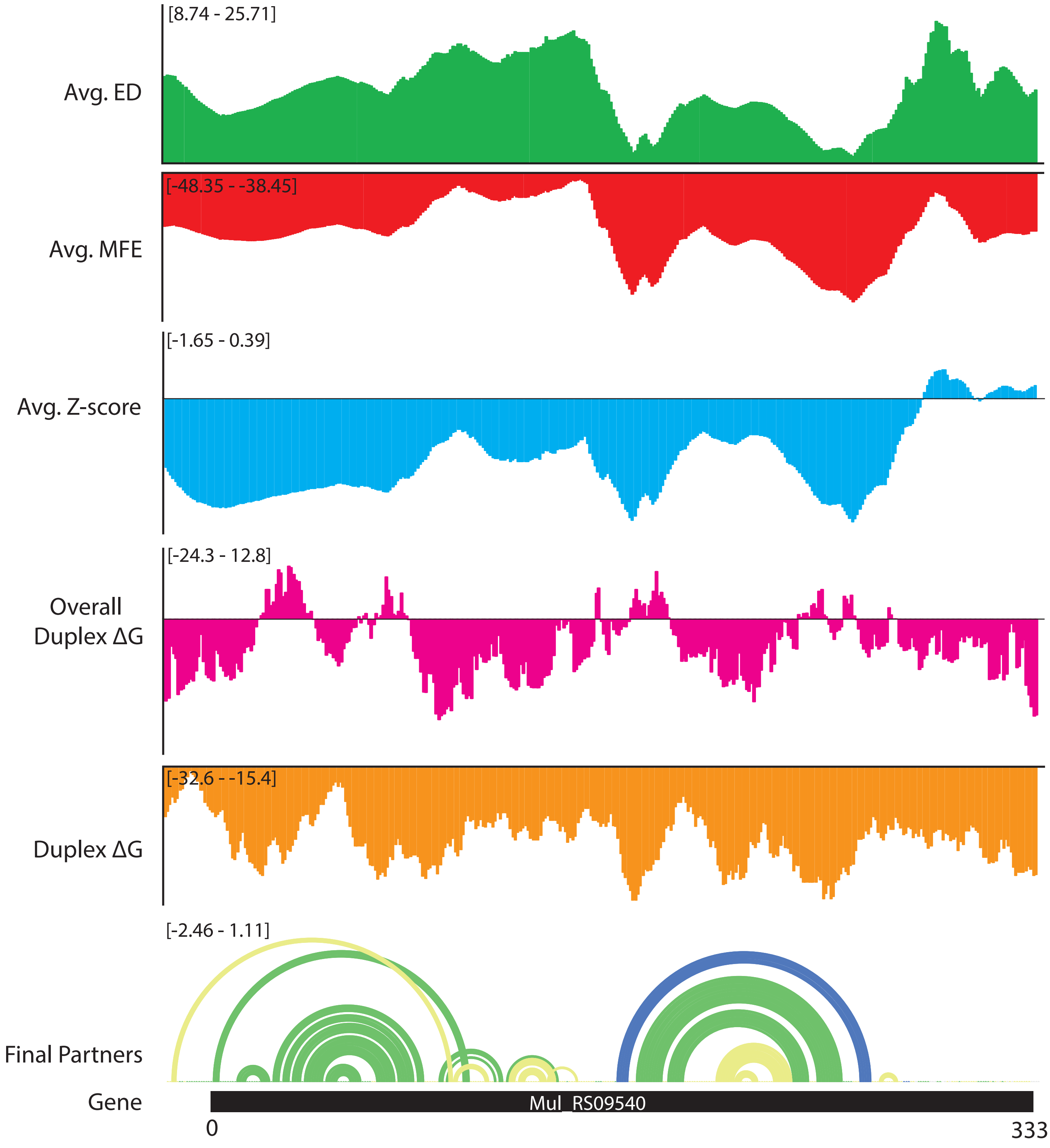

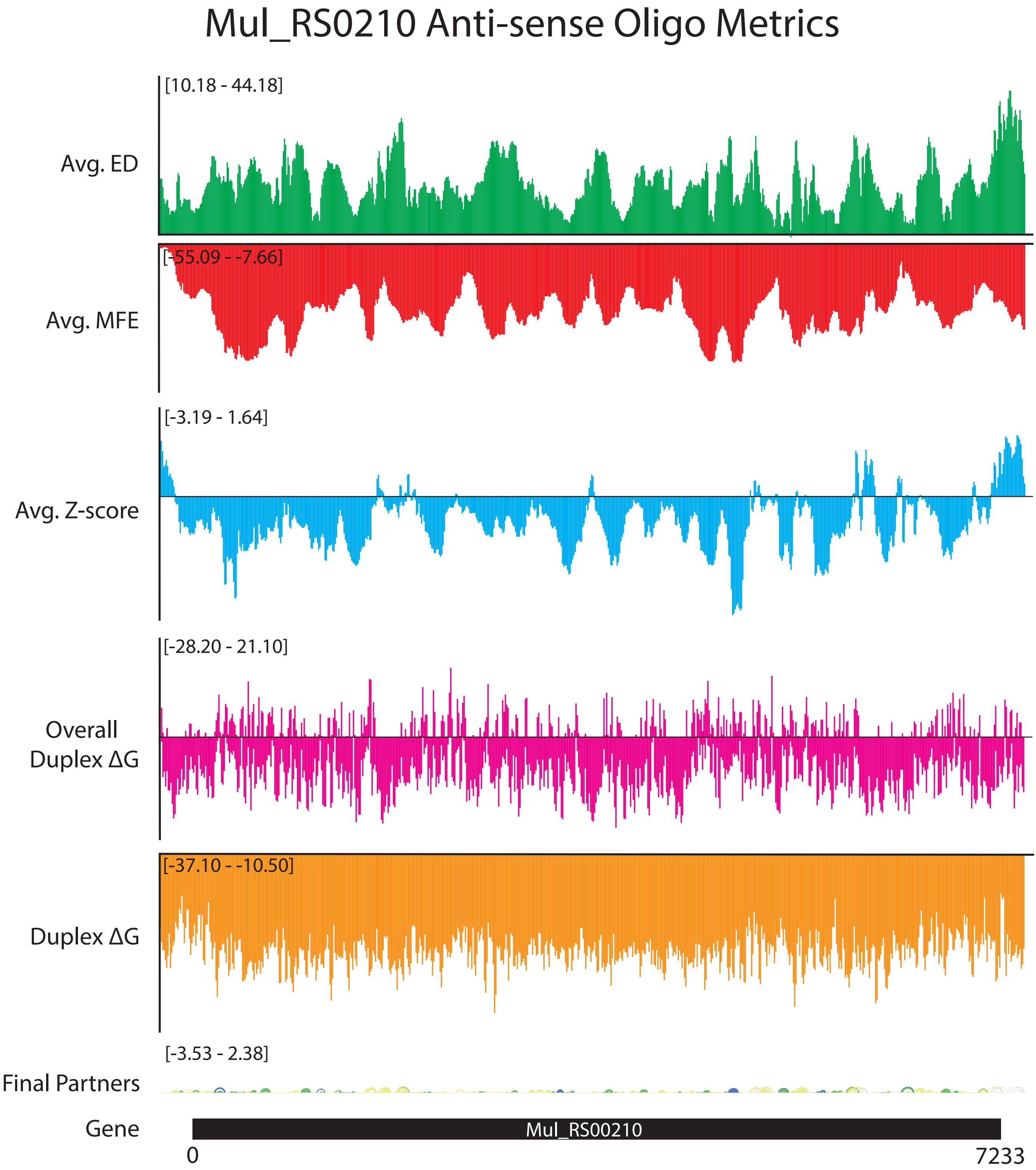

Mul_RS0210 Anti-sense Oligo Metrics
Avg. ED
[10.18 - 44.18]
Avg. MFE
[-55.09 - -7.66]
Avg. Z-score
[-3.19 - 1.64]
Overall
Duplex ΔG
[-28.20 - 21.10]
Duplex ΔG
[-37.10 - -10.50]
Final Partners
[-3.53 - 2.38]
Gene
Mul_RS00210
0
7233

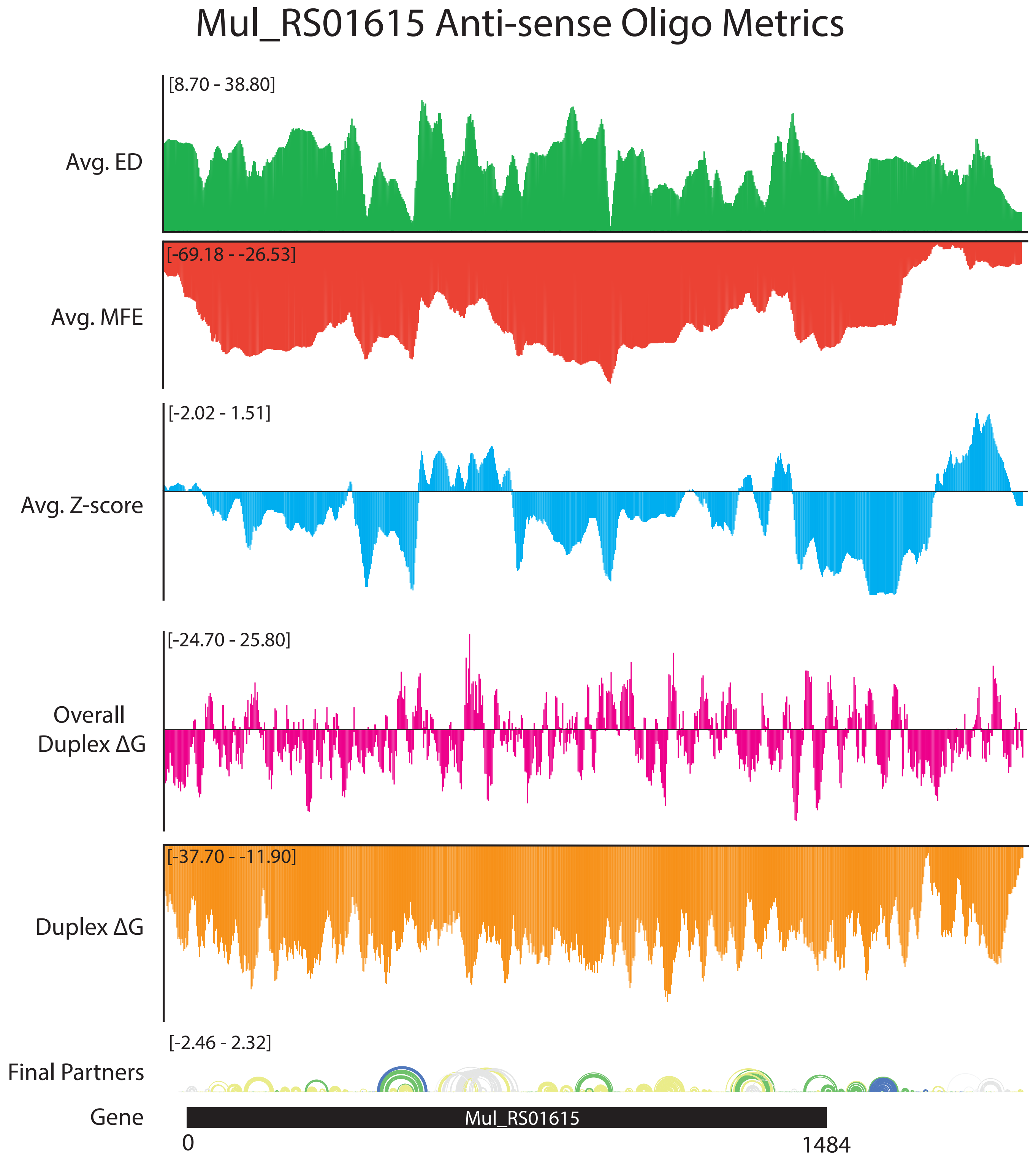
Mul_RS01615 Anti-sense Oligo Metrics
[8.70 - 38.80]
Avg. ED
[-69.18 - -26.53]
Avg. MFE
[-2.02 - 1.51]
Avg. Z-score
[-24.70 - 25.80]
Overall Duplex ΔG
[-37.70 - -11.90]
Duplex ΔG
[-2.46 - 2.32]
Final Partners
Gene
Mul_RS01615
0
1484
