# Supplementary material for: Analysis of key genes in Mycobacterium ulcerans reveals conserved RNA structural motifs and regions with apparent pressure to remain unstructured
Source: Front Trop Dis. Author manuscript; Available in PMC 2023 Mar 30. (PMC10062443; doi:10.3389/fitd.2022.1009362)
Supplement: All Zipped Supplemental Material — SUPPLEMENTARY FIGURE 1 All Mul_RS04200 ScanFold results. Figure showing ScanFold results for Mul_RS04200 including z-score, MFE, ED, base pair diagram, gene cartoon, and 2D model of the structure with a z-score <−2. SUPPLEMENTARY FIGURE 2 All Mul_RS09540 ScanFold results. Figure showing ScanFold results for Mul_RS09540 including z-score, MFE, ED, base pair diagram, gene cartoon, and 2D model of the structure with a z-score <−2. SUPPLEMENTARY FILE S1 M. ulcerans genomic data used in IGV-ScanFold. This file contains the M. ulcerans bacterial genome fasta, virulence plasmid fasta, and their associated gff3 genome annotations. SUPPLEMENTARY FILE S2 All cm-builder covariation data. This file contains all the data required to run cm-builder, all the output files generated by INFERNAL and R-Scape, and results of power analysis. SUPPLEMENTARY FILE S3 OligoWalk and 18-mer ScanFold bar charts. This file contains the OligoWalk and 18-mer partitioned ScanFold data as bar charts overlaid against the gene cartoon for all six genes studied. SUPPLEMENTARY FILE S4 OligoWalk and 18-mer ScanFold raw data. This file contains the raw output data from OligoWalk and in-house script for portioning 18-mer ScanFold data for all six genes of interest. SUPPLEMENTARY FILE S5 All ScanFold-Scan data. This file contains two folders with results from mono- and dinucleotide shuffling for each gene studied. These folders contain the raw ScanFold-Scan output data such as per nucleotide MFE, ED, z-score, input, and output fasta files, and out file. SUPPLEMENTARY FILE S6 All ScanFold-Fold data. This file contains two folders with results from mono- and dinucleotide shuffling for each gene studied. These folders contain the raw ScanFold-Fold output data such as the log file, base pair track, final partners data, all dot bracket files, all CT files, extracted structures gff3 file, and the global VARNA 2D model. [file NIHMS1880345-supplement-All_Zipped_Supplemental_Material.zip › Supplementary Data/Supplementary File S6.zip!/Supplementary File S6/ScanFold-Fold_ReadMe.docx]

**ScanFold-Fold Files ReadMe**

**Log.txt:** This file contains the raw output of ScanFold-Fold. This will give the list of all base pairs predicted for each nucleotide, the number of windows each are observed in, and the average metrics for each.

**IGV.bp:** This file contains data that generates the arc diagram in IGV. It describes the connections between nucleotides and the header contains information such as the color used to depict the arc. It corresponds to the base paired nucleotides that are recurring across low z-score analysis windows during ScanFold-Fold.

**Final_partners.txt:** This file contains the genomic position, paired nucleotides, average MFE, average z-score, and average ED of the final base pairs predicted by ScanFold-Fold.

**ExtractedStructures.gff3:** This file contains a list of extracted sequences and structures from ScanFold-Fold that have z-scores greater than or equal to -2, the start and end coordinates, refolded structures using -2 z-score constraints, MFE, z-score, and ED. Viewable on IGV.

**DBNstructures.txt:** This file contains the sequences and predicted structures in dot bracket notation for the input sequence. This includes a global refold with -2 z-score constraints, all structures without a z-score filter, all -1 z-score structures, and all -2 z-score structures.

**Scanfoldvarna:** This file contains the 2D model generated by VARNA using the ScanFold-Fold predicted structures. This model is annotated with average per nucleotide z-score values.

**Dbnfile1.dbn:** This file contains the sequence and all predicted structures from the input sequence

**Dbnfile2.dbn:** This file contains the sequence and all predicted structures with a z-score of -1 or less from the input sequence.

**Dbnfile3.dbn:** This file contains the sequence and all predicted structures with a z-score of -2 or less from the input sequence.

**-2filter.dbn:** This file contains the sequence and refolded structures found in the scanned sequence using -2 z-score constraints from ScanFold-Fold.

**Nofilter.ct:** This file is a connectivity file containing the sequence and all structures found from the input sequence. Viewable on IGV.

**-1filter.ct:** This is a connectivity file containing the sequence and all structures found with a z-score of -1 or less from the input sequence. Viewable on IGV.

**-2filter.ct:** This is a connectivity file containing the sequence and all structures found with a z-score of -2 or less from the input. Viewable on IGV.
